# Gene Therapy: A Revolutionary Step in Treating Thalassemia

**DOI:** 10.3390/hematolrep16040064

**Published:** 2024-10-21

**Authors:** Jhancy Malay, Rasha Aziz Attia Salama, Ghania Shehzad Alam Qureshi, Ali Raafat Ali Ahmed Ammar, Gayatri Janardhan, Maryam Safdar, Hesham Amin Hamdy Elshamy

**Affiliations:** 1 Department of Pediatrics, RAK Medical and Health Sciences University, Ras Al Khaimah 11127, United Arab Emirates; 2 Department of Community Medicine, RAK Medical and Health Science University, Ras Al Khaimah 11127, United Arab Emirates; rasha.aziz@rakmhsu.ac.ae; 3 RAK College of Medical Sciences, RAK Medical and Health Sciences University, Ras Al Khaimah 11127, United Arab Emirates; ghania.19901026@rakmhsu.ac.ae (G.S.A.Q.); ali.19901007@rakmhsu.ac.ae (A.R.); gayri.19901025@rakmhsu.ac.ae (G.J.); maryam.23908002@rakmhsu.ac.ae (M.S.); 4 Department of Surgery, RAK Medical and Health Sciences University, Ras Al Khaimah 11127, United Arab Emirates; hesham@rakmhsu.ac.ae

**Keywords:** beta thalassemia major, Cooley’s anemia, gene addition, gene editing, gene modification, Mediterranean Anemia, transfusion-dependent beta thalassemia

## Abstract

Beta thalassemia is an inherited blood disorder that results in inefficient erythropoiesis due to genetic mutation that leads to the reduction or absence of the hemoglobin beta-globulin protein. Approximately 8.5% of UAE residents suffer from β-thalassemia, a significant health and financial problem. The treatment options available for β-Thalassemia major are limited and associated with a wide range of complications. β-thalassemia gene therapy is emerging as a potential novel treatment option that eliminates the complications caused by the current long-term treatment modalities and the associated economic burden. This paper reviews the scientific literature related to emerging gene therapy for β-Thalassemia by analyzing all the articles published from January 2010 to December 2023 in the English language on Databases like PubMed, Scopus, ProQuest, and CINAHL. The use of gene therapy has demonstrated promising outcomes for a permanent cure of β-Thalassemia. To conclude, gene therapy is an innovative solution. It demonstrates a promising future, but does come with its own setbacks and is something that must be tackled in order to revolutionize it in the medical world. FDA-approved ZYNTEGLO is a potentially one-time curative treatment for β-Thalassemia. Although cutting-edge, its use is limited because of the high cost—a price of USD 2.8 million per patient.

## 1. Introduction

Thalassemia is a group of autosomal recessive inherited blood disorders. It can be divided into two forms: Alpha and Beta-thalassemia. The cause of the blood defects is gene mutations leading to low levels and/or malfunctioning/absence of the hemoglobin α and β globin proteins, respectively [1]. Based on the varying degrees of severity, Beta thalassemia exists in three phenotypic forms: thalassemia major, thalassemia intermedia, and thalassemia minor. Individuals with thalassemia major, also known as transfusion-dependent β-thalassemia (TDT), usually present within the first two years of life with severe anemia, requiring red blood cell transfusions regularly [2]. The level of Hb might be <7 g/dL and Hb F < 90%. Individuals with untreated or poorly transfused thalassemia major usually present with growth retardation, pallor, jaundice, poor musculature, hepatosplenomegaly, leg ulcers, development of masses from extramedullary hematopoiesis, and skeletal changes that result from the expansion of the bone marrow to compensate for the loss in RBCs [3].

Traditionally, β-thalassemia has been more common in the Mediterranean, Middle East, and Southeast Asia. Approximately 8.5% of UAE residents suffer from B-thalassemia, a significant health and financial problem [4]. An estimated AED 1.2 million is reported as the cost of treatment for TDT patients aged 16 and under. As a result of the high prevalence of thalassemia carriers in the country, a marriage between two carriers poses a 25% risk of having a child with TDT [5].

The main treatment modalities for transfusion-dependent thalassemia include regular blood transfusions, iron chelation therapy, and medications like luspatercept (Ribosyl) and hydroxyurea [6]. Regular transfusion therapy commonly leads to iron overload-related complications including endocrine complications (growth retardation, failure of sexual maturation leading to infertility, diabetes mellitus, and insufficiency of the parathyroid, thyroid, pituitary, and adrenal glands), dilated myocardiopathy, liver fibrosis, and cirrhosis. Additionally, secondary viral infections are also relatively common. Furthermore, the search for a compatible donor proves to be another barrier [7].

The only potentially curative option for thalassemia major is allogeneic hematopoietic-cell transplantation, but owing to risks of graft rejection, graft-versus-host disease, and other treatment-related toxic effects, transplantation is mainly reserved for young children with an HLA-identical sibling donor [8]. Therefore, gene therapy is currently being evaluated as a new option in patients with β-thalassemia. The aim of this narrative review is to analyze novel treatment options especially gene therapy for transfusion-dependent beta thalassemia.

## 2. Materials and Methods

We conducted an extensive literature search in four major databases: PubMed, ProQuest, Scopus, and CINAHL. Articles published in English from January 2010 to December 2023 were included in the review. The search strategy was based on the following query: (Gene therapy OR Gene Editing OR Genetic Modification OR Gene Addition) AND (Beta Thalassemia Major OR Transfusion dependent thalassemia OR Cooley’s Anemia OR Mediterranean Anemia). In addition, Medical Subject Headings (MeSH) terms were utilized to refine the search: Gene Therapy OR Genetic Therapies AND Thalassemia Major (beta-Thalassemia Major).

The initial search identified a total of 13,116 articles. Articles were included if they were published in English, focused on gene therapy or genetic modification techniques for beta thalassemia major and addressed transfusion-dependent beta thalassemia. Articles were excluded if they were not directly related to gene therapy or genetic modification for beta thalassemia major, not published within the specified date range, editorials, or opinion pieces without original research data, single case reports and animal studies. Not all but some recent clinical trials were included to narrate the ongoing research on gene therapy.

Data extraction was performed independently by seven authors to ensure accuracy and reduce bias. Discrepancies in data extraction were resolved through discussion among the authors until a consensus was reached.

## 3. Results

**Figure 1:**
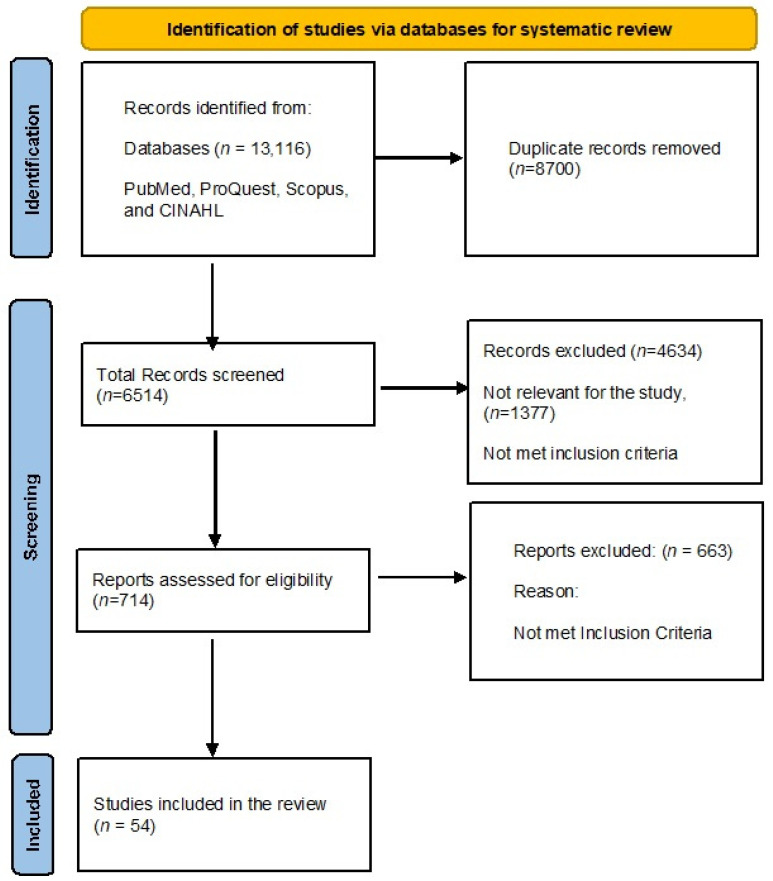
PRISMA Flow diagram of articles searched.

Out of a total of 13,116 articles, 8700 duplicates and 4634 articles that did not meet inclusion criteria were excluded. Finally, 54 articles, followed by 4 hand-picked articles, were included for a total of 58 articles in this narrative review. These studies examined various conventional therapies and gene therapy techniques aimed at treating transfusion-dependent beta thalassemia (Figure 1).

## 4. Discussion

### 4.1. Treatment Modalities

The management of beta thalassemia major presents a nuanced interplay of established and evolving treatment strategies, each tailored to mitigate the complex manifestations of the disorder. Central to this paradigm are transfusion and chelation therapies, pioneering in anemia management and mitigating iron overload. Moreover, conventional treatment utilizes splenectomy and hemoglobin F inducers to promote hemoglobin levels and effective erythropoiesis [8,9]. However, the pursuit of curative interventions has led to the forefront of hematopoietic stem cell transplantation, offering a potential respite for afflicted individuals, particularly in the pediatric population [10]. Novel strategies aimed at reducing iron absorption have represented an area of interest. Apo-transferrin (apo-Tf), hepcidin agonists, JAK2 inhibitors, Tmprsso inhibitors, and activin receptor II (ActRII) ligand traps hold promise for improving iron overload management, averting splenomegaly and thrombosis, and decreasing the need for transfusions in individuals with ß-thalassemia [9,11]. Upcoming scientific advancements are paving the way for promising treatments like gene therapy, ushering in a new future for the management of beta thalassemia major [11].

#### 4.1.1. Conventional Therapy

##### Transfusion Therapy with Iron Chelation Therapy (ICT)

Patients diagnosed with β-thalassemia major (BTM) necessitate regular blood transfusions, complemented by appropriate iron chelation therapy (ICT), throughout their lifespan. These transfusions serve to address chronic anemia, prevent bone deformities, support normal growth and activity levels, and enhance the overall quality of life (QoL) [5,12]. By providing fresh, normal red blood cells (RBCs), transfusions rectify anemia and suppress ineffective erythropoiesis, consequently averting hepatosplenomegaly and restricting bone marrow hyperplasia [5,8]. The advent of ICT has markedly improved life expectancy in BTM patients, attributed to heightened awareness among healthcare providers and patients, safer blood processing guidelines, enhanced techniques for iron overload assessment, and the availability of oral ICT options [13,14]. Notably, leukoreduced-packed RBCs sourced from voluntary donors are preferred, with a minimum hemoglobin content of 40 g recommended [12]. The decision to initiate transfusions hinges upon anemia presence, clinical symptoms, and comorbidities like organ dysfunction. Untreated BTM typically results in fatality before adolescence, while transfusion-supported patients can anticipate healthy longevity [5,7,12]. However, transfusions carry risks such as acute (e.g., hemolytic reactions) and delayed adverse events (e.g., transfusion-transmitted infections, iron overload-related complications). To mitigate the iron accumulation’s detrimental effects, timely initiation of ICT, particularly oral agents like deferiprone and deferasirox, is imperative. Although ICT constitutes a significant portion of medical expenses, its judicious use significantly reduces the burden and costs associated with thalassemia complications and blood transfusions [7,12,15,16,17].

##### Splenectomy

In thalassemia major and intermedia, splenomegaly stemming from severe hemolysis can be mitigated through regular blood transfusions, yet hypersplenism may develop in children aged 5–10 years. In such cases, splenectomy becomes a consideration, especially when transfusion requirements exceed 200–220 mL RBCs/kg with 70% hematocrit or 250–275 mL/kg packed RBCs with 60% hematocrit annually [18,19,20]. Despite the benefits, including decreased transfusion needs and improved hemoglobin levels, splenectomy poses risks, such as sepsis, venous thromboembolism, pulmonary hypertension, and leg ulcers [5,12]. Nevertheless, it remains an option for select patients facing complications like hypersplenism or worsening anemia not responsive to transfusion therapy. Thrombocytosis post-splenectomy necessitates vigilance and, if persistent, management with aspirin to mitigate thrombotic complications [20,21,22]. Screening for accessory splenic tissue and ongoing assessment for transfusion requirements are imperative in managing splenectomies in thalassemia patients [17,21,23]. Moreover, preoperative immunization against meningococcal and pneumococcal infections, followed by antimicrobial prophylaxis, aids in infection prevention, adding another layer of care [5,19].

##### Hemoglobin F Inducers

HbF inducers present a promising approach in thalassemia treatment by increasing γ-globin production, a β-like globin molecule capable of sequestering excess α-chains, thus countering the α/β-chain imbalance characteristic of the condition and enhancing effective erythropoiesis [9]. Notably, hydroxyurea’s mechanisms include γ-globin induction, inhibition of ribonucleotide reductase, and modulation of gene expressions related to apoptosis, cell cycle, and erythropoiesis [24,25]. Hydroxyurea (or hydroxycarbamide), extensively studied as an HbF inducer in β-thalassemia, has shown promising hematological improvements, with long-term safety deemed satisfactory. However, conflicting data primarily from single-arm trials or retrospective cohort studies warrant large-scale randomized controlled trials before widespread adoption in management [26].

While hydroxyurea is approved for reducing transfusion dependency in sickle cell disease, its efficacy in β-thalassemia remains uncertain [25]. Guidelines suggest considering hydroxyurea for specific β-thalassemia patients, such as those who are alloimmunized or have specific genetic polymorphism [27]. However, close monitoring of response is crucial, with alternative treatments considered if necessary [12,24]. Other potential fetal Hb inducers, including 5-azacytidine and decitabine, have been explored, yet clinical evidence for their use remains limited [5,7,28].

#### 4.1.2. Novel Modalities

##### Gene Therapy

The main objective of gene therapy is to increase the synthesis of β- or ɣ-globin, thereby reducing the amounts of unattached α-globin chains, to reestablish the alpha/non-alpha globin ratio in RBCs. By doing so, RBC hemolysis and ineffective erythropoiesis are prevented, leading to prolonged RBC lifespan with functional haemoglobin-carrying erythrocytes. This results in the correction of anaemia and reduces the need for multiple transfusions [29].

Gene therapy advancements, particularly using CRISPR-Cas9 technology, show promise in correcting the defective b-globin gene and increasing HbF levels, potentially offering functional cures for beta-thalassemia patients. Novel gene therapy techniques, including CRISPR-Cas9, present significant prospects for treating beta-thalassemia by reducing the risk of iron overload, minimizing ineffective red blood cell production, and potentially offering cures for suitable patients in the future. Monitoring the safety, efficacy, and long-term effects of gene therapy treatments is essential to assess their durability, benefit–risk ratio, and potential expansion to a broader patient population [30].

Autologous gene modification treatment requires several steps:

- Extraction of hematopoietic stem and progenitor cells (HSPCs): HSPCs, which can undergo self-renewal and multilineage differentiation, are found in the bone marrow. These cells express CD34 and are responsible for producing blood cells throughout a person’s lifetime. Collection of the cells can be performed either from the bone marrow or the peripheral blood [31,32]. The conventional method of collecting autologous bone marrow is invasive and yields insufficient CD34+ HSPCs. Patients with B-thalassemia have an increased accumulation of erythroid precursors in their bone marrow, which can hinder the harvesting of an adequate dose of stem cells. Therefore, it is necessary to mobilize the stem cells [29,31].
- Mobilization and apheresis: Mobilization of cells from bone marrow to peripheral blood is carried out using the recommended combination of granulocyte-colony stimulating factor (G-CSF) and plerixafor before collection by apheresis [31]. A recent study shows that the combined use of G-CSF and plerixafor provides an adequate yield of stem cells [33].
- Myeloablation: Myeloablation of the bone marrow is the next step and is performed to create enough space for the gene-modified HSPCs to be reinserted, ensuring efficient engraftment. The recommended myeloablative agent is busulfan since the dose can be tailored and modified for each patient depending on the response to the drug [33].
- Modified HSPCs infusion: The final step is the infusion of the cryopreserved genetically modified HSPCs, using 5% dimethylsulfoxide (DMSO) solution, intravenously after it meets all the release criteria (sterility, viability, purity, and vector copy number [VCN] for gene-insertions), and minimum cell dose criteria (>2–3 × 10^6^ CD34+/kg) [33]. Gene therapy for TDT can be divided into two parts based on the method used, gene insertion and gene editing [34].

##### Gene Insertion

Gene insertion is a technique in which functional copies of genes, in this case, the β or ɣ-globin-producing genes, are introduced to the autologous HSPCs by using viral vectors, usually retro or lentiviruses, that carry the functional gene alongside its regulatory elements and insert it into the patient’s genome. The insertion of the vectors into the cells could either be in-vivo or ex-vivo [35].

Some of the risks associated with this approach are that the viral vector and insertion in an area near a proto-oncogene can stimulate uncontrolled proliferation leading to cancer development. For example, Hargrove et al. found that insertional dysregulation of cellular genes could be caused by lentiviral vectors [36]. Hence, extensive preclinical testing is necessary and follow-up for these patients is required for 15 years to detect any adverse events.

Fortunately, this method of gene therapy is showing great promise. For example, Thompson et al. performed a clinical trial in which 22 patients with TDT received a functional copy of the β-globin gene using a lentiviral vector into autologous CD34+ hematopoietic stem cells. The results showed that 15 out of the 22 patients (68%) achieved transfusion independence. Moreover, those patients also showed improved hemoglobin production and hematological parameters along with reduced iron overload. As for adverse effects, they were mild and resolved without sequelae. Finally, the author concluded that gene therapy has great potential to treat TDT, but further studies are warranted to confirm its long-term safety and efficacy [29].

One more study investigated the effectiveness of various mobilization methods for obtaining CD34+ cells from 31 thalassemic patients for globin gene therapy. Patients were treated with hydroxyurea combined with G-CSF, G-CSF alone, Plerixafor, or a combination of Plerixafor and G-CSF. The CD34+ cells were transduced with a β-globin lentivector and analyzed for transduction efficiency and engraftment potential in a xenogeneic model. Results showed that Plerixafor+G-CSF mobilized cells had the highest β-globin expression relative to vector copy number and achieved superior early engraftment rates. Overall, Plerixafor+G-CSF proved to be the most effective approach for generating a suitable graft for thalassemia gene therapy, facilitating higher yields and better expression of β-globin [37].

Another study by Marktel et al. in which three adults and six children were treated with a different vector (GLOBE) showed significant hematopoietic recovery with reduced transfusion dependence in the adults along with complete discontinuation of transfusion in three out of four pediatric patients. The authors proposed that younger age is associated with a better outcome; nonetheless, the trial is still in progress [38].

Another clinical trial is the Northstar-3 trial (HGB-212; NCT03207009) which is a phase 3 study assessing LentiGlobin gene therapy efficacy and safety in patients with TDT and either β^0^ or β^+^ IVS-I-110 mutations on both HBB alleles. The study included 18 patients, of whom 16 (89%) achieved transfusion independence for at least 12 months after treatment, maintaining sustained hemoglobin levels with a median of 10.1 g/dL. Abdominal pain, leukopenia, neutropenia, thrombocytopenia, and pyrexia were some of the adverse events related to LentiGlobin therapy; however, overall, the therapy was well-tolerated in the majority of participants [39].

##### Gene Editing

Gene editing is a novel technique that allows scientists to make precise and accurate modifications and edits in different sequences of the DNA and enables modifications to specific sites on the genome. This is different from gene insertion which is semi-random and lacks exactness.

The various types of targeted nucleases operate on a similar principle. First, they need to recognize a certain part of DNA and bind to it effectively and precisely. Secondly, certain regions of the DNA have to be “cut” or cleaved. The various methods can differ in how they bind to DNA, their nature, modularity of recognition, size of the recognition domain, and the resultant cleavage [38].

Some examples include meganucleases, zinc finger nuclease (ZFN), transcription activator-like effector nucleases (TALENS), and clustered regularly interspaced short palindromic repeats (CRISPR)-associated nuclease 9 (Cas9). An issue that could arise is unintended edits in other parts of the genome or what is known as “off-target” activity [40]. SOX 6 gene is a transcription factor that represses gamma-globin expression which in turn reduces gamma globin and increases synthesis of defective beta-globin in cases of thalassemia. Disruption of this gene has been shown to up-regulate gamma-globin expression in adult RBC cells and this could compensate for the loss of beta-globin in beta-thalassemia. Using this principle, a study performed by Laleh et al. used three different single-guide RNA CRISPR/cas9 to disrupt the SOX 6 gene in defective RBC cells in order to increase gamma-globin expression. The investigators suggest that this approach could be a potential approach for gamma-globin reactivation, but further studies are needed to know the degree of improvement that it might provide and its safety [40,41,42].

The introduction of CRISPR/Cas technology has revolutionized genome editing, allowing for precise targeting of DNA sequences in HSCs to correct mutations associated with β-thalassemia. Two primary DNA repair mechanisms, non-homologous end joining (NHEJ) and homology-directed repair (HDR), are utilized, with NHEJ being the more efficient pathway for inducing therapeutic changes. Current applications focus on using NHEJ to increase fetal hemoglobin (HbF) levels, which can effectively treat severe β-thalassemia mutations [43].

Recent genome-wide association studies (GWASs) have identified B-cell Leukemia/Lymphoma 11A (BCL11A) as a key regulator of fetal hemoglobin (HbF) expression that represses HbF and controls its transition to HbA. This finding has significant implications, as inactivation of BCL11A has been shown to induce the reactivation of fetal globin expression in human cells [44]. Genetic modifiers, such as BCL11A and KLF1, play crucial roles in regulating fetal globin gene expression and switching from g-globin to b-globin expression. Understanding genetic modifiers and mechanisms associated with beta-thalassemia is crucial for developing targeted therapies and improving patient outcomes [45].

In one study, thirty-three months after receiving lentiviral β-globin gene therapy, an adult patient with severe βE/β0-thalassemia, who had relied on monthly blood transfusions since childhood, has been transfusion-independent for the last 21 months. The patient’s blood hemoglobin levels are maintained between 9 and 10 g/dL, with about one-third consisting of vector-encoded β-globin. The primary therapeutic benefit appears to stem from a dominant myeloid-biased cell clone, where the integrated vector activates HMGA2 transcription in erythroid cells, leading to increased production of a truncated HMGA2 mRNA that is resistant to degradation by let-7 microRNAs. The clonal dominance associated with the treatment’s success might be coincidental and random or could be due to a previously benign cell expansion linked to HMGA2 gene dysregulation in stem or progenitor cells [46].

The trials CLIMB THAL-111 [40] and CLIMB SCD-121 [47] used CTX001, which is an autologous cellular drug that contains CRISPR Cas9 ribonucleoprotein, targeting the BCL11A erythroid-specific enhancer region to disrupt it and produce more gamma-globin. Two patients were included in this study: one with TDT every year for 2 years and the other with SCD along with a history of two or more severe vaso-occlusive episodes per year. After receiving myeloablative therapy using busulfan, both patients were infused with CTX001 and were followed for 21 months.

The first patient with TDT showed significant improvement after only 18 months in which her hemoglobin increased from 9.0 to 14.1 g/dL (93% fetal hemoglobin) and became transfusion dependent. The only serious adverse events were pneumonia and veno-occlusive liver disease which resolved. The second patient with SCD also showed an improvement in her hemoglobin levels 15 months after the intervention, from 7.2 to 12.0 g/dL, and had no new vaso-occlusive episodes. The major adverse event was sepsis which was resolved.

This clinical trial has shown optimistic results regarding the use of CTX001 gene therapy in the editing of BCL11A. Also, it showed that CTX001 results in stable engraftment, high levels of fetal hemoglobin expression, and the elimination of vaso-occlusive episodes or the need for transfusion. Even so, the long-term effect of CTX001 remains unknown, and the ability to generalize the findings to other TDT or SCD patients is not clear, these initial results show great promise for the future of gene editing [32].

Lentiviral systems, known for their safety and efficacy in delivering therapeutic genes, have been adapted for gene-editing applications. However, a significant challenge in in vivo gene addition and editing is the need for larger quantities of vectors due to inefficient targeting of hematopoietic stem cells (HSCs) and their uptake by phagocytic cells. Enhancing vector specificity for HSCs could mitigate this issue. Additionally, the patient’s immune response may neutralize the gene delivery vectors, particularly due to preexisting antibodies against certain adeno-associated virus (AAV) serotypes. Current in vitro assays often favor short-term progenitors and may underestimate editing efficiencies in true engrafting HSCs, leading to competition from unedited cells. Therefore, studies in non-human primates are preferred to better evaluate the long-term engraftment potential of gene-edited cells, as xenograft mouse models may inaccurately overestimate contributions from progenitor cells [48].

Emerging data from the CLIMB THAL-111 and CLIMBSCD-121 trials, presented at the American Society for Gene and Cell Therapy Annual Meeting, indicate that a single dose of the investigational gene therapy achieved transfusion independence in 95% of beta-thalassemia patients [32].

##### Gene Therapy Genotoxicities

While lentiviral vectors (LVVs) have a lower risk of oncogenic activation compared to γ-retroviral vectors, there are still concerns regarding potential genotoxic effects, including the activation of oncogenes. Instances of adverse events, such as the development of a dominant HSC clone linked to LVV insertion, highlight the need for ongoing monitoring of long-term safety in gene therapy patients. Research is focused on understanding the integration profiles of LVVs and the potential for off-target effects, necessitating long-term follow-up for patients receiving gene therapy [43,49].

##### Genetic Activation of HbF Expression for β-Thalassemia

The regulation of HbF expression involves repressor proteins like BCL11A, and recent studies have explored disrupting these proteins to enhance HbF production in patients with β-thalassemia. Clinical trials, such as the CLIMB THAL-111 study, have shown promising results, with a majority of participants achieving transfusion independence after receiving gene-modified therapies. Other trials, like the THALES trial, have faced challenges in sustaining HbF levels, indicating the need for more effective long-term genetic modifications in HSCs. Base editing offers a novel approach to induce HbF by creating new binding motifs in the γ-globin promoter, potentially leading to more effective treatments than traditional methods. A new clinical trial by Beam Therapeutics aims to utilize adenine base editing to modify the γ-globin promoter, which could be adapted for β-thalassemia if successful [43].

##### Zynteglo

Betibeglogene autotemcel (beti-cel), Zynteglo by Bluebird Bio-Inc) is the only autologous hematopoietic stem cell-based (HSC) gene therapy approved by the Food and Drug Administration (FDA) in August of 2022. It is a curative therapy, as an intravenous infusion, indicated for adult and paediatric patients suffering from transfusion-dependent B-thalassemia [50]. The basis of the therapy is to introduce functional copies of the beta gene in order to produce functional adult Hb. The suspension consists of the patient’s own mobilised CD34+ hematopoietic stem cells that are transduced ex-vivo with the BB305 lentiviral vector. The vector encodes for the β-globin (βA-T87Q) gene. Prior to administration of the infusion, myeloablative conditioning is required [51].

The transplanted gene aims to correct the imbalance of the haemoglobin chains, normalize adult haemoglobin levels, and thereby eliminate the need for frequent blood transfusions. A 2022 phase-3 open label trial, evaluating the efficacy and safety of beti-cel, reported that 20 of the 23 patients achieved transfusion independence. For the patients who did not show transfusion independence, it was observed that there was a reduction of 30% and 26% in transfusion frequency [52]. A study assessed the health-related quality of life (HRQoL) of patients who had undergone the phase-3 trial. It reported improvement in all domains of HRQoL (physical, psychological, social, environmental) in the patients who received Zynteglo [13,39].

The most common adverse effects were found to be GIT disturbances (vomiting, nausea, diarrhoea and abdominal pain, constipation, low appetite), fever, mucositis, febrile neutropenia, musculoskeletal pain, cough, pigmentation disorder, headache, rash, itch, hair loss and nose bleeds. Serious adverse reactions (<3%) included fever, thrombocytopenia, liver venoocclusive disease, febrile neutropenia, neutropenia, and stomatitis. No fatalities were reported. The conditioning agents used may be the reason for the side effects observed [53]. Considering that Zynteglo has not been studied in pregnant and lactating female patients and the risks surrounding myeloablative conditioning, the therapy is not indicated in pregnancy and lactation. Patients are advised to use an effective contraceptive method for at least 6 months after receiving Zynteglo as the effect on contraception is not sufficiently studied. Similarly, the effect on fertility has insufficient data, thus patients may take necessary precautions, if appropriate. Although indicated for the pediatric and adult populations, the safety and efficacy of the treatment are not established in children less than 4 years old and in older adults. Health-care professionals involved in the treatment are advised to monitor patients for possible hypersensitivity reactions, thrombocytopenia, bleeding, and failure of neutrophil engraftment. Annual screening for hematological malignancies is also recommended [54].

While Zynteglo is certainly a ground-breaking advancement in the world of gene therapy, there are concerns related to the affordability of the therapy because of its high cost of around 2.8 million US dollars [52]. This major drawback was the reason for its withdrawal from the European market after receiving approval from the European Medicines Agency (EMA) in 2019 [55]. Nonetheless, the one-time beti-cel therapy has been found to be cost-effective as compared to the standard of care in a study conducted in the United States [56].

##### Perspectives and Future

Current treatment strategies for beta-thalassemia patients have improved quality of life, but come with risks and limitations due to the complex nature of the disease. Gene therapy approaches hold promise for providing functional cures for beta-thalassemia patients by augmenting HbF levels, but further research is needed to determine the safest and most effective therapies. Monitoring the safety profile and long-term effects of gene therapy treatments is crucial for assessing their overall benefit–risk ratio and expanding their availability to a broader patient population [45,57].

Novel gene therapy approaches offer potential functional cures for transfusion-dependent beta-thalassemia (TDT) patients by addressing the root cause of the disease and aiming for durable RBC transfusion independence with improved quality of life. Characterizing the safety profile of novel conditioning therapies is crucial for expanding the availability of gene therapy treatments to a broader set of patients and assessing the overall benefit–risk ratio of the treatment. Monitoring the persistence of gene-edited alleles and assessing the impact of gene insertion using lentiviral vectors in long-term follow-up studies is essential to evaluate the durability of the treatment effect [58,59].

The FDA’s approval of betibeglogene autotemcel marks a significant milestone in gene therapy for β-thalassemia, with ongoing advancements in genome editing technologies promising further improvements. Future developments may include more efficient gene therapy processes, such as in vivo therapies and less toxic conditioning regimens, to enhance patient access and treatment outcomes. Collaboration among stakeholders is essential to address the challenges of high costs and regulatory complexities, ensuring equitable access to these innovative therapies

In the present review, we observed that conventional therapies, such as regular blood transfusions and chelation therapy, typically show efficacy within a few weeks to months after initiation. Whereas for the gene therapy approach, significant and sustained improvements, such as transfusion independence, were documented around 21 months after the lentiviral β-globin gene transfer [46].

## 5. Conclusions

The treatment options available for β-Thalassemia major are limited and associated with a wide range of complications. The main therapy to maintain healthy levels of hemoglobin is blood transfusion. The maximum range of RBCs that can be transfused without giving a spike increase in the volume of blood is only 15–20 mL/kg. While well-treated patients commonly reach adulthood, their life expectancy is greatly diminished due to complications of iron overload. Regular blood transfusions result in the accumulation of iron within the reticuloendothelial system as the iron excretory mechanisms of the body are compromised. The deposited iron harms endocrine glands, and blood vessels, enhances the risk of liver carcinoma, and cirrhosis and can lead to diabetes and infertility. Prolonged blood transfusion products may also pave the way for pathologic hepatitis. Finding a compatible blood donor is a significant barrier in itself. Other solutions include bone marrow transplant, iron chelation therapy and splenectomy, each coming with its own set of challenges

Gene therapy is an innovative solution. On 19 August, Betibeglogene autotemcel (ZYNTEGLO), onetime cell-based gene therapy was officially FDA-approved. It is a potentially curative treatment to correct the globin chain imbalance, thus potentially improving the production of normal hemoglobin, erythropoiesis and chronic anemia. Treatment with beti-cel is expected to result in a significant clinical benefit and lower the patient’s high lifetime expenses. These advantages stem from increased life expectancy, higher quality of life, reduced complications, and the avoidance of chelation treatment and ongoing transfusions.

Although cutting edge, it is high in cost, subsequently limiting availability. With its list of unlikely adverse effects came the biggest adverse effect: a 2.1 million USD price tag per person. This is only the cost of the medication; the price of hospitalization, etc., remains separate. Establishing the durability of treatment, incidence of risks associated with the therapy, use in special populations, and cost-effectiveness requires long-term follow-up and research.

## Data Availability

The data will be available freely on search databases with keyword search.
